# Evidence of Horizontal Gene Transfer of 50S Ribosomal Genes *rplB*, *rplD*, and *rplY* in *Neisseria gonorrhoeae*

**DOI:** 10.3389/fmicb.2021.683901

**Published:** 2021-06-10

**Authors:** Sheeba Santhini Manoharan-Basil, Jolein Gyonne Elise Laumen, Christophe Van Dijck, Tessa De Block, Irith De Baetselier, Chris Kenyon

**Affiliations:** ^1^Department of Clinical Sciences, Institute of Tropical Medicine Antwerp, Antwerp, Belgium; ^2^Laboratory of Medical Microbiology, Vaccine and Infectious Disease Institute, University of Antwerp, Antwerp, Belgium; ^3^Department of Medicine, University of Cape Town, Cape Town, South Africa

**Keywords:** 50S ribosomal proteins, azithromycin resistance, *rplY* (L25), *rplD* (L4), *rplB* (L2), HGT in *Neisseria gonorrhoeae*

## Abstract

Horizontal gene transfer (HGT) in the *penA* and multidrug efflux pump genes has been shown to play a key role in the genesis of antimicrobial resistance in *Neisseria gonorrhoeae*. In this study, we evaluated if there was evidence of HGT in the genes coding for the ribosomal proteins in the *Neisseria* genus. We did this in a collection of 11,659 isolates of *Neisseria*, including *N. gonorrhoeae* and commensal *Neisseria* species (*N. cinerea*, *N. elongata*, *N. flavescens*, *N. mucosa*, *N. polysaccharea*, and *N. subflava*). Comparative genomic analyses identified HGT events in three genes: *rplB*, *rplD*, and *rplY* coding for ribosomal proteins L2, L4 and L25, respectively. Recombination events were predicted in *N. gonorrhoeae* and *N. cinerea*, *N. subflava*, and *N. lactamica* were identified as likely progenitors. In total, 2,337, 2,355, and 1,127 isolates possessed L2, L4, and L25 HGT events. Strong associations were found between HGT in L2/L4 and the C2597T 23S rRNA mutation that confers reduced susceptibility to macrolides. Whilst previous studies have found evidence of HGT of entire genes coding for ribosomal proteins in other bacterial species, this is the first study to find evidence of HGT-mediated chimerization of ribosomal proteins.

## Introduction

*Neisseria gonorrhoeae* is a sexually transmitted pathogen that causes the disease gonorrhea (Tapsall et al., 2009; Quillin and Seifert, 2018). Treatment guidelines typically recommend dual antimicrobial therapy, including ceftriaxone and azithromycin (Unemo et al., 2020). The rapid emergence of antimicrobial resistance to azithromycin in numerous populations threatens this treatment approach and has recently led to the dropping of azithromycin from treatment guidelines in countries such as the United States (Martin et al., 2016; Wan et al., 2018; St Cyr et al., 2020). Azithromycin [9-deoxo-9a-aza-9a-methyl-9a-homoerythromycin], a 15-membered macrolide, binds to the bacterial large (50S) ribosomal subunit, which consists of 23S rRNA, 5S rRNA, and ribosomal proteins. It interferes with protein synthesis via various mechanisms, including inhibiting the transpeptidation/translocation step, blocking the 50S peptide exit tunnel, and causing ribosomes to release incomplete peptides (Douthwaite and Champney, 2001; Champney and Miller, 2002; Unemo and Shafer, 2014).

The molecular mechanisms that cause azithromycin resistance in *N. gonorrhoeae* include an alteration of the drug target either by methylation of a single adenine in 23S rRNA (*rrl*) or chromosomal mutations (Roberts et al., 1999; Chisholm et al., 2010). Three such mutations have been found in domain V of the 23S rRNA: C2597T, A2045G, and A2046G (Chisholm et al., 2010; Pham et al., 2020). In addition, overexpressed efflux pumps [multidrug efflux pump (MtrCDE), MacAB, and *mef*-encoded pumps] may reduce azithromycin susceptibility (Unemo and Shafer, 2014). The MtrCDE efflux pump plays an essential role in azithromycin resistance. The *mtr* locus contains three genes (*mtrC* – *mtrD* – *mtrE*) within an operon that forms a MtrCDE and contains genes for a transcriptional repressor (*mtrR*) upstream of *mtrCDE* (Golparian et al., 2014). Mutations upstream of *mtrC*, including those within the MtrR binding region and mutations in the promoter region and mosaic-like sequences within the *mtrD* gene, contribute to reduced susceptibility to azithromycin (Hagman and Shafer, 1995; Zarantonelli et al., 1999; Shafer, 2018).

The above well-characterized resistance-associated mutations (RAMs) are, however, unable to explain all the variation in gonococcal susceptibility to macrolides (Ma et al., 2020). A point mutation in the 50S ribosomal protein, L4 (G70D), has been implicated in macrolide resistance in *N. gonorrhoeae* and other bacteria (Gregory and Dahlberg, 1999; Tait-Kamradt et al., 2000; Ma et al., 2020). Recently, a genome-wide association study conditioned on the known resistance mechanisms, with experimental validation, confirmed that the G70D mutation resulted in reduced susceptibility to azithromycin. In addition, the study identified other L4 mutations at amino acid positions 68 (G68D, G68C), 69 (T69I), and 70 (G70S, G70A, G70R, and G70duplication) associated with reduced azithromycin susceptibility (Ma et al., 2020). These mutations are all situated at the end of the L4 loop close to the macrolide binding site (Gregory and Dahlberg, 1999; Tait-Kamradt et al., 2000; Ma et al., 2020). In a recent *in vitro* study investigating the molecular pathways to high-level azithromycin resistance in *N. gonorrhoeae*, we found mutations in 50S ribosomal proteins L4, L22, and L34 that play a prominent role in the genesis of azithromycin resistance (Laumen et al., 2020). Similar mutations in L4 and L22 have been shown to cause macrolide resistance in several other bacterial species (Gregory and Dahlberg, 1999; O’Connor et al., 2004; Halling and Jensen, 2006; Diner and Hayes, 2009).

Horizontal gene transfer (HGT) occurs via conjugation, transformation and transduction. In *Neisseria*, natural transformation is the primary mode of HGT which occurs with remarkable frequency and efficiency and is dependent on type IV pilus complex (Biswas et al., 1977; Koomey, 1998; Chen and Dubnau, 2004). DNA uptake sequence (DUS), 5’-GCCGTCTGAA-3’ is required for efficient transformation and is both strain and strand specific in *N. gonorrhoeae* (Goodman and Scocca, 1988; Smith et al., 1999; Ambur et al., 2007; Duffin and Seifert, 2010).

Both chromosomal mutations and HGT have been shown to play an important role in the genesis of aminocyclitol (spectinomycin), macrolide (azithromycin), and cephalosporins (ceftriaxone and cefixime) resistance (Ilina et al., 2013; Grad et al., 2016). HGT has been particularly important in the genesis of resistance at the *rpsE*, *mtrCDE*, and *penA* loci (Bowler et al., 1994; Ilina et al., 2013; Wadsworth et al., 2018; Yahara et al., 2018). We hypothesized that HGT in the 50S ribosomal genes might play a similar role in generating macrolide resistance in *N. gonorrhoeae*. Several studies suggest that ribosomal proteins that are both essential to function (e.g., L12 and L2) and not essential to function are amenable to HGT in other bacterial species (Weglöhner et al., 1997; Ühlein et al., 1998; Garcia-Vallvé et al., 2002; Miyazaki and Tomariguchi, 2019). As such, we assessed if there was any evidence of HGT in the gonococcal 50S ribosomal genes in *N. gonorrhoeae*. We considered all *N. gonorrhoeae* isolates (11,659 genomes available from Pathogenwatch at the time of data collection during April 2020). We set-out by analyzing these species that may have undergone HGT in the 50S ribosomal genes with the Neisseria commensal species (*N. cinerea*, *N. elongata*, *N. flavescens*, *N. mucosa*, *N. polysaccharea*, and *N. subflava*).

## Materials and Methods

### Data Extraction

As of April 2020, all 27 collections of *N. gonorrhoeae* whole genome sequences (WGS) that were available from the global platform for genomic surveillance of microbial pathogens, Pathogenwatch^1^, were downloaded. These comprised a total of 11,993 genomes. Plausibility checks were carried out to remove duplicates (*n* = 349), and in total, 11,659 WGS [*N. gonorrhoeae* (*n* = 11,644) from pathogenwatch and commensal *Neisseria* spp. (*n* = 15) from NCBI (GenBank)] were used in the study (Tables 1, 2 and Supplementary Table 1). The following outcomes were extracted from the metadata: azithromycin MIC, azithromycin SIR (susceptible/intermediate/resistant), collection label, country, genogroup, MLST, NG-STAR type, SNPs (23S and *mtrR*), and year. When the azithromycin MIC was only reported as S (susceptible) or R (resistant), we assigned an azithromycin value of 0.25 mg/L for S, and 2 mg/L for R isolates. Also, we defined isolates as azithromycin susceptible, intermediate, and resistant if their MICs were <0.5 mg/L, ≥0.5 and <1 mg/L, and ≥1 mg/L, respectively. The isolates were divided into three distinct eras: “pre-antibiotic” (pre-1950s), “golden” (1950–1970s), and “post-modern” (1980-twenty-first century) following the topology of Golparian et al., 2020.

**TABLE 1:** Pathogenwatch collections used in this study.

Collections	Years	Countries	Number (n) of genomes	PMID
Alfsnes et al., 2020	2016–2017	Norway	816	32213251
Osnes et al., 2021	2015–2018	Norway	133	NA
Schmerer et al., 2020	2016	United States	324	32071056
Cehovin et al., 2018	2010–2015	Coastal Kenya	112	29701830
Grad et al., 2016	2000–2013	United States	1035	27638945
Ezewudo et al., 2015	1982, 2005–2008	Canada (*n* = 17) and Chile (*n* = 1)	18	25780762
Wind et al., 2017	2002–2012	Netherlands (*n* = 21) and Suriname (*n* = 2)	23	28510723
Thomas et al., 2019	2014–2016	United States	644	30788502
Grad et al., 2014	2009–2010	United States	216	24462211
Lan et al., 2020	2011 and 2016	Vietnam	227	32068837
Golparian et al., 2020	1928–2013	Denmark	192	32013864
Chisholm et al., 2016	2015	United Kingdom	14	26601852
Ryan et al., 2018	2012–2016	Ireland	42	29882175
Kwong et al., 2016	2004–2015	Australia (*n* = 30), New Zealand (*n* = 18)	48	28348871
Kwong et al., 2018	2006–2014	Australia	75	29247013
Buckley et al., 2018	2012 and 2014	Australia	92	29367612
Fifer et al., 2018	2004–2017	Scotland (*n* = 28), United Kingdom (*n* = 72)	100	29523496
Didelot et al., 2016	1995–2004	United Kingdom	194	27353752
Demczuk et al., 2016	1997–2014	Canada	200	26935729
Sánchez-Busó et al., 2019	1979–2013	Several countries*	395	31358980
Demczuk et al., 2015	1989–2013	Several countries*	168	25378573
Yahara et al., 2018	2011–2015	Japan	245	30063202
Town et al., 2020	2013–2016	United Kingdom	1288	31978353
De Silva et al., 2016	2004–2015	United Kingdom	1783	27427203
Williamson et al., 2019	2017	Australia	2179	31488838
Lee et al., 2018	2014–2015	New Zealand	376	29182725
European Centre for Disease Prevention and Control, 2015	2013	Several countries*	1054	NA

*cf. Supplementary Table 1.

**TABLE 2:** Reference genomes used in this study.

Organism	Number of isolates	GeneBank accession/Strain
*N. cinerea*	1	NZ_LS483369
*N. elongata*	4	NZ_CP007726
		NZ_CP031252
		NZ_CP031255
		NZ_LS483435
*N. flavescens*	1	NZ_CP039886
*N. lactamica*	4	NC_014752
		NZ_CP019894
		NZ_CP031253
		NZ_LR590477
*N. mucosa*	2	CP028150
		NZ_CP020452
*N. polysaccharea*	1	NZ_CP031325
*N. subflava*	2	NZ_CP031251
		NZ_CP039887
*N. gonorrhoeae*	20	CP012028.1 (Strain 35/02)
		AE004969.1 (Strain FA1090)
		CP012026.1 (Strain FA19)
		CP012027.1 (Strain FA6140)
		CP003909.1 (Strain MS11)
		CP001050.1 (Strain NCCP11945)
		LT591897.1 (Strain WHO_F)
		LT591898.1 (Strain WHO_G)
		LT591908.1 (Strain WHO_K)
		LT591901.1 (Strain WHO_L)
		LT591904.1 (Strain WHO_M)
		LT591910.1 (Strain WHO_N)
		LT592146.1 (Strain WHO_O)
		LT592157.1 (Strain WHO_P)
		LT592159.1 (Strain WHO_U)
		LT592150.1 (Strain WHO_V)
		LT592163.1 (Strain WHO_W)
		LT592155.1 (Strain WHO_X)
		LT592161.1 (Strain)WHO_Y
		LT592153.1 (Strain WHO_Z)

### Gene by Gene Analysis

**FIGURE 1:**
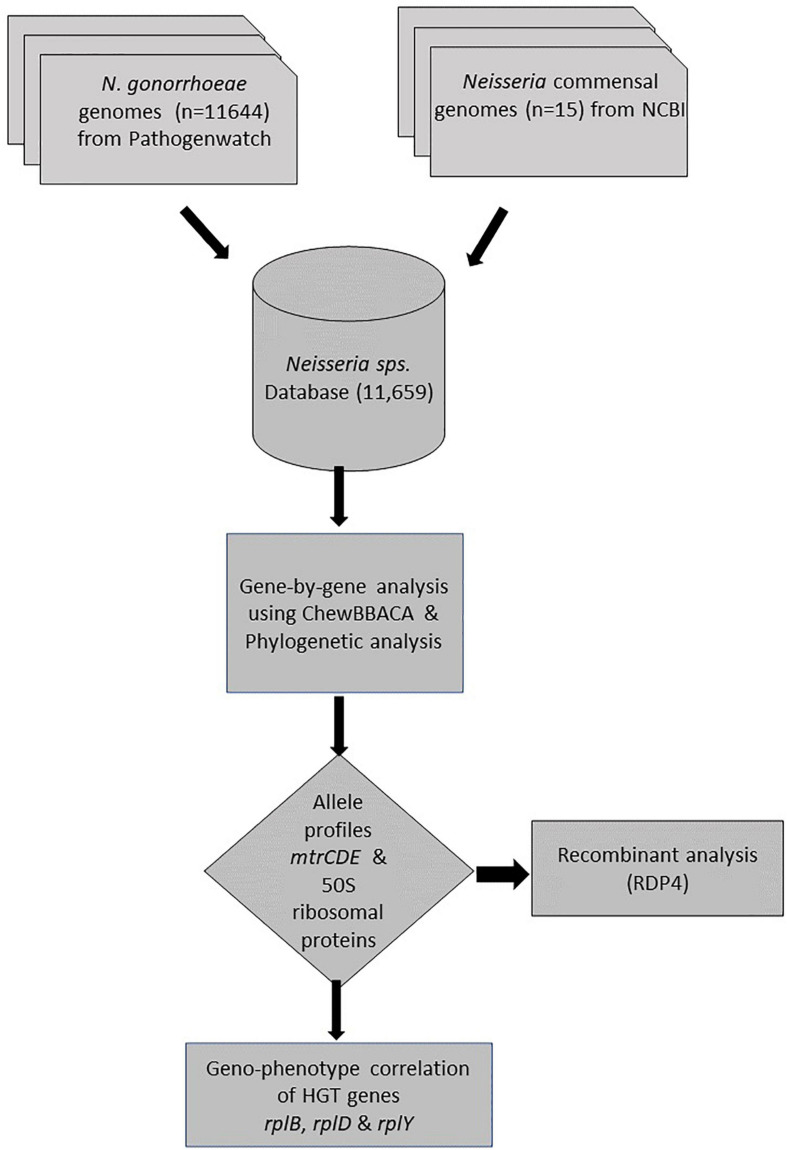
Flowchart describing study design and analysis.

A gene-by-gene approach on whole-genome sequences (WGS; *n* = 11659) followed by recombination analysis was carried out (Figure 1). A study-specific *Neisseria* scheme was created from 35 complete *Neisseria* genomes [*N. gonorrhoeae* reference genomes (*n* = 20) and commensal *Neisseria* sps. (*n* = 15), Table 2] using the Blast Score Ratio Based Allele Calling Algorithm (chewBBACA; Silva et al., 2018). The complete genome of *N. gonorrhoeae* FA1090 was used to create a training file using Prodigal, which was used in subsequent steps (Hyatt et al., 2010). Firstly, coding sequences (CDS) for each genome were defined and compared in a pairwise manner to generate a single FASTA file containing the selected CDS. Secondly, AlleleCall implemented in chewBBACCA was carried out and followed by filtering out paralogous alleles, thus creating the wgMLST profiles used to define the cgMLST profiles. The SchemaEvaluator option allows for multiple sequence alignments of the alleles of each locus using MAFFT (Katoh et al., 2002) and the construction of a neighbor-joining tree using ClustalW2 (Larkin et al., 2007). The UniProtFinder^2^ was used to retrieve the functional information of the CDS. 50S ribosomal and *mtrCDE* genes were selected based on the UniProt identifier, and the corresponding multiple sequence alignments of the alleles and neighbor-joining trees were extracted from the schema evaluator. The trees and the corresponding metadata were visualized using microreact (Argimón et al., 2016).

### Allelic Profiling

Alleles were identified for the ribosomal genes. The FASTA files of the ribosomal genes that were generated from cgMLST were used as input sequences in MEGAX (Kumar et al., 2018). The CDS were translated, and all the variable amino acid sites that were singleton or parsimony-informative were exported. If a non-synonymous substitution (variant) site was present only in a commensal, it was excluded from variant calling and further analysis. The presence or absence of a variant for each site was denoted as “0” and “1,” respectively. The geometric mean (GM) AZM MIC was calculated for each allele.

### Recombination Analysis

To identify the donors of mutation-harboring ribosomal genes (*n* = 23) in *N. gonorrhoeae* isolates, complete genomes of commensal *Neisseria* spp., including *N. cinerea*, *N. elongata*, *N. flavescens*, *N. lactamica*, *N. mucosa*, *N. polysaccharea*, and *N. subflava* were analyzed. The nucleotide alignment of each ribosomal gene derived from cgMLST were screened for the presence of recombination events using the Recombination Detection Program (RDP4) program (Martin et al., 2015). Recombinant events supported by at least two of the seven algorithms implemented in RDP software: RDP, GENECONV, Bootscan, Maxchi, Chimera, SiSscan, and 3Seq were used with default settings except for window size that was increased to 60 nt in RDP, to 120 in MaxChi and Chimera, and to 500 in BootScan and SiScan (Lemey et al., 2009; Gomez-Valero et al., 2011). Neighbor-joining phylogenetic trees were constructed using 1,000 bootstrap replicates (Salminen et al., 1995). We defined the minor parent as the one contributing the smaller fraction of the recombinant, and the major parent as the one contributing the larger fraction of the recombinant (Martin et al., 2015). Additionally, sequences in the order of 5’ to 3’: 100 bp upstream of *rplD* (621 bp), *rplW* (321 bp), *rplB* (834 bp) and 100 bp downstream of *rplB* (length −1,971 nt) from *Neisseria* commensals (*n* = 15) and subset of *N. gonorrhoeae* sequences, ECDC isolates (*n* = 1,054) were aligned and followed by recombination analysis using RDP4 program.

### Statistical Analysis

Statistical analyses were performed using JMP Pro V14.0.0 (SAS Institute, NC, United States) and XLSTAT (Statistical and data analysis solution, New York, United States). MIC variables were log_2_-transformed for use as continuous outcome variables (Ma et al., 2020). Differences between variant and wildtype were analyzed by the Mann–Whitney test. Only mutations present in >10 isolates were used in these analyses. To evaluate the correlation between multiple pairs of variables (SNPs) and to assess the correlations between phenotypic and genotypic patterns of resistance to azithromycin, statistical correlation tests including Kendall’s tau-b and Spearman’s rank correlation were used (Elhadidy et al., 2020). Differences in categorical variables were evaluated by Chi-square/Fischer exact tests. A *p-*value of <0.001 was considered statistically significant.

## Results

### *Neisseria gonorrhoeae* Isolates Used in This Study

A total of 11,993 *N. gonorrhoeae* genome sequences were available at Pathogenwatch. Of these, 349 were duplicates represented in more than one study, and thus 11,644 unique *N. gonorrhoeae* genomes were included in the study. The isolates were from 68 countries (Supplementary Figure 1A) and the period of sampling spanned over 90 years (Supplementary Figure 1B). A total of 456 different sequence types (STs) characterized the available population. The most frequent ST was ST1901 (*n* = 1,549). Twenty-nine AZM high-level resistant (AZM-HLR, MIC ≥ 256 mg/L) isolates were detected and belonged to the following ST types: 1,579 (*n* = 2), 1,580 (*n* = 8), 1,901 (*n* = 7), 7,822 (*n* = 3), 7,823 (*n* = 1), 9,363 (*n* = 4), 10,314 (*n* = 1), 10,899 (*n* = 1), 10,931 (*n* = 1), and 12,039 (*n* = 1). Further details pertaining to distribution of MLST and AZM MICs are provided in Supplementary Table 2.

### Prevalence of Known RAMs

**FIGURE 2:**
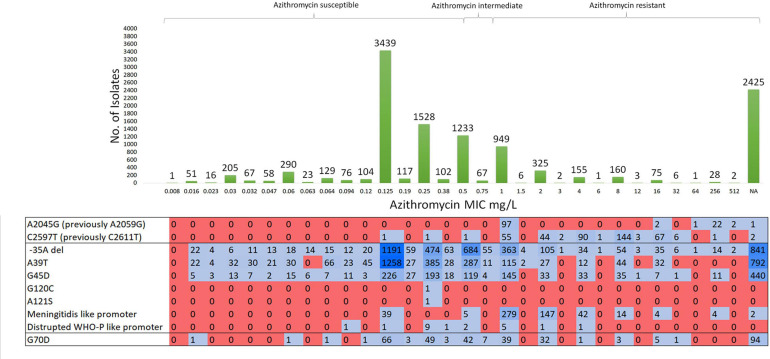
Molecular characterization of susceptible, intermediate-, and resistant-azithromycin *N. gonorrhoeae* isolates and their known resistance associated mutations. Total number of isolates – 11644.

The MICs of azithromycin and the molecular determinants of known RAMs of the *N. gonorrhoeae* isolates analyzed in this study are summarized in Figure 2. Of the 11,644 *N. gonorrhoeae* isolates, AZM MIC value was not available for 2,520 (21.64%) isolates; 6,124 (52.59%) isolates were susceptible, 3,000 (25.76%) isolates were non-susceptible to azithromycin, including 1,593 (13.68%) resistant, and 1,407 (12.08%) intermediate isolates. The MIC’s of azithromycin ranged from 0.008 to 512 mg/L (Figure 2).

Mutations in domain V of 23S rRNA were detected in 540 (4.63%) isolates. Azithromycin-resistant *N. gonorrhoeae* isolates contained A2045G (*n* = 124) and C2597T (*n* = 416) substitutions with MICs of azithromycin ranging from 1 to 512 mg/L. Of note, two isolates with C2597T mutation were susceptible to azithromycin with an MIC of 0.125 mg/L (ERR3325414) and 0.25 mg/L (SRR8560311).

Mutations in the *mtrR* promoter (-35A deletion) were present in 4,125 (35.42%) isolates. Of these, AZM MIC was not available for 835 (20.24%) isolates, and 1,934 (46.8%), 744 (18.03%), and 612 (14.83%) of these isolates were susceptible, intermediate, and resistant to AZM, respectively. Non-synonymous SNPs (A39T, G45D, G120C, and A121S) in MtrR were present in 4,660 (40.02%) isolates (2,509 azithromycin susceptible, 406 azithromycin intermediate, and 513 azithromycin resistant). The G120C and A121S amino acid substitutions of MtrR were each observed in only one susceptible isolate. *N. meningitidis*-like or WHO-P-like *mtrR* promoter mutations were present in 557 isolates with a high proportion of resistant isolates (*n* = 490) containing the *N. meningitidis*-like *mtrR* promoter mutation. Non-synonymous mutations in *rplD* at positions G68C/D/V and G70A/D/R/S were found in 2,760 *N. gonorrhoeae* isolates which were all significantly associated with higher AZM MICs (Supplementary Table 3 and Supplementary Figure 3).

### Gene-by-Gene Analysis Reveals the Diversity of 50S Ribosomal Variants

**FIGURE 3:**
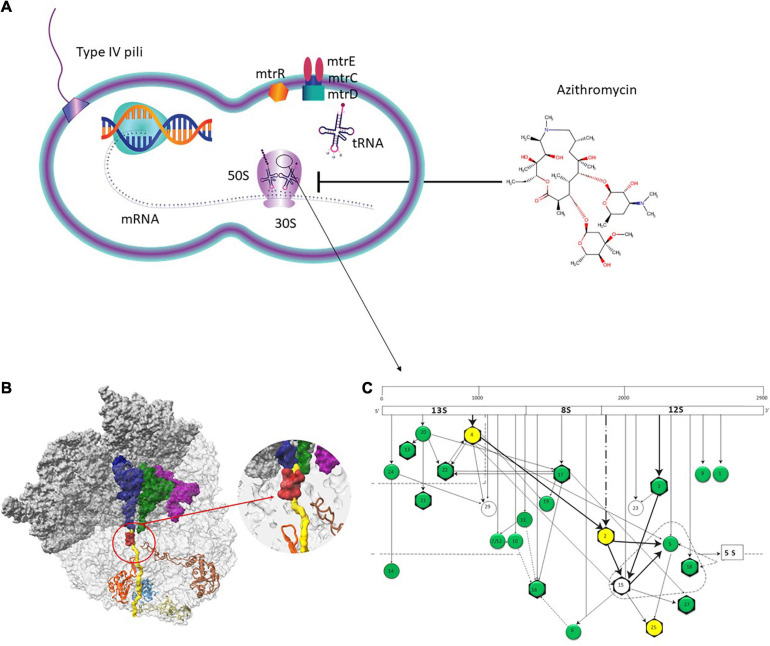
Azithromycin action pathway. **(A)** Azithromycin targets the protein synthesis of bacteria by binding to the 50S subunit of the bacterial ribosome near the polypeptide exit tunnel. Efflux pumps, such as MtrCDE protect *N. gonorrhoeae* from antimicrobials. **(B)** The ribosome nascent chain tunnel environment in *Staphylococcus aureus*. Left: The 70S *S. aureus* (SA_WT) ribosome where the large subunit is shown in light gray and the small subunit is shown in dark gray (PDBID 5TCU). The A-site, P-site, and E-site docked tRNA molecules (from PDBID 5JTE) are shown in blue, green, and magenta, respectively. The surface of a nascent chain within the peptide exit tunnel is shown in yellow. Bound macrolide (erythromycin) is shown in red, uL4, uL22, uL23, and uL24 are shown in brown, orange, teal, and khaki, respectively. Right: zoom into the macrolide binding site at the upper tunnel. Figure reproduced with permission from Halfon et al. (2019). This image is distributed under the terms of the Creative Commons Attribution Non-Commercial 4.0 International license (CC-BY-NC), a copy of which is available at https://creativecommons.org/licenses/by/4.0/. **(C)** Assembly map of 50S subunit of *E. coli* ribosomes. The main fragments of 23S rRNA (13S, 8S, and 12S) are indicated (top bar) as well as the ribosomal proteins (circles). The arrows denote the direction of the dependence of ribosomal proteins. Proteins in the dotted triangle are important for mediating the binding of 5S rRNA to 23S rRNA. Proteins colored in green and yellow were analyzed in the present study. Proteins with evidence of horizontal gene transfer (HGT) are colored in yellow. L31 and L31-Type B proteins are not depicted. Figure adapted with permission from Rohl and Nierhaus (1982).

**FIGURE 4:**
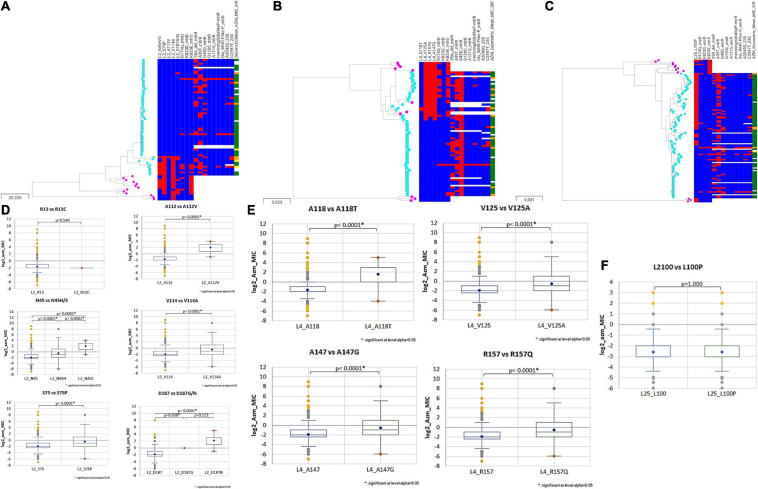
Phylogenetic trees of **(A)** *rplB* **(B)** *rplD* **(C)** *rplY* based on *Neisseria* sps. (*N. gonorrhoeae*, *N. lactamica*, *N. subflava*, *N. cinerea*, and *N. mucosa*) cgMLST allelic profiles determined by the chewBACCA software. The tree was created with Microreact. Cyan node denotes *N. gonorrhoeae*, and pink nodes denote *Neisseria* commensals. Blue and red color denote the absence and presence of non-synonymous SNPs, respectively. The geometric mean azithromycin MIC SIR profiles are denoted as green, yellow, and orange, respectively. **(D–F)** Statistical significance between non-synonymous SNPs and wildtype log_2_ AZM MIC distributions as assessed by Mann–Whitney *U* Test. The line inside the box marks the median. The upper and the lower hinges corresponds to the 25th and 75th percentiles. Statistical significance between variants and wildtype MIC distributions are depicted, *p* < 0.0001 in all cases, except *rplA* (R13) wildtype and variant (R13S), and *rplY* (L100) wildtype and variant (L100P).

The 50S subunit of the *N. gonorrhoeae* genome (FA1090) consists of 36 ribosomal proteins, designated L1 to L36 (Figure 3C). Alleles were identified for the following 23 ribosomal genes: *rplA*, *rplB, rplC*, *rplD*, *rplE*, *rplF*, *rplL*, *rplI, rplK*, *rplM*, *rplN*, *rplP*, *rplQ*, *rplS*, *rplT*, *rplV*, *rplU*, *rplX*, *rplY*, *rpmA*, *rpmB*, *rpmE*, and *rpmE2* which encodes L1, L2, L3, L4, L5, L6, L7/L12, L9, L11, L13, L14, L16, L17, L19, L20, L21, L22, L24, L25, L27, L28, L31, and L31-type B ribosomal proteins, respectively. A total of 336 non-synonymous mutations were determined for the 23 ribosomal genes (Table 3). A number of the newly described mutations had raised GM AZM MICs compared to the wild type: T129I mutation in L1 (*rplA*, *n* = 158, Supplementary Figure 3A), R157Q mutation in L4 (*rplD*, *n* = 2,335, Figure 4E), and A66V in L17 (*rplQ*, *n* = 47, Supplementary Figure 3B).

**TABLE 3:** Amino acid diversity of 50S ribosomal genes.

S.NO	Gene/protein name	No. of alleles	No. of isolates with no alleles assigned	Non-synonymous SNPs
1	*rplA* (L1)	57	1	S14F, E16G, D24Y, T69I, T72I, R74L, T79A, A84T, A90V, D99N, A101T, E103K, T129A/I, P133S, N139S, A152V, K154T, T165A, A173V, A204T, S216C, T227I
2	*rplB* (L2)	74	4	A2V, S10P, R13C, V20I, A28V, A31T, N45H/S, H60R, S75P, A112V, V114A, T129A, A160V, D187G/N, S201G, N261H
3	*rplC* (L3)	78	11	R8H, T13A, A32V, T36I, T43A, Q55R, R60C, A66T, G67E, H68Y, A70V, G79S, A95V, D97N, T115S/I, K126T, A132V/T, S140F, H143Y, M151V, A152V, A165T, T171I, T174A, A185V, A199S, R207C, P208S, V212A, A214L
4	*rplD* (L4)	74	14	V12I, G14D, H33R/Y, V36A, N37S, R53C, P63T, G68C*/D*/V, T69I*, G70A*/D*/R*/S*, G70 duplication*, T77I, S78P, P80S, W82R, A88S/T, A118T, V125A, T130I, A147G, R157Q, L158F, A177V, S184G, R187H, I193V, A199V
5	*rplE* (L5)	36	4	R3Q, E15G, G21D, V28F, R59K, G62D, Q63R, R64K, F77S, P84L, P109T, S121A, G124D, M130V, T155I, T162I, L172F, F173L, K174N, P176R, F177S
6	*rplF* (L6)	32	4	N7K, E16K, H38R, V53I, S57N, T67S, A68G, V76I, K86R, I90M, I102T, T134A, S146A, V168L, A714V, K176T
7	*rplL* (L7/L12)	45	2	A2V, I3V, A39V, V40A, A43V, P45del, Del48A, Del48P, del49G, del49A, G50D/A/Del, A51V, D55A, A56T, T60I, A69V, A69G, I102L, S107F, A111T, A120V, A120E, E126G
8	*rplI* (L9)	63	2	D17N, A26S, A33V, G34S, R38H, A42V, R50C, R51H, A56V, A59V, A67V, D73N, T78I, T94A, A105V, P117S, P120L, V129L, A148S
9	*rplK* (L11)	43	1	K4R, A27V, A41V, T68I, A76S, L80F, A83S/V, S90G, T95A, N96S, A104V, P115L, T118A, D123A, A124E, A124V, A125T, T128A, A133V, M136I, D139G
10	*rplM* (L13)	52	1	A20V, L25S, T30A, S34H, H47N, I54V, I55V, I57V, A59V/T, R63C, A67V, H77Y, Y85C, R87L, E91K, D94E, G98D, E120G, M118V, H130Y, K143R
11	*rplN* (L14)	32	3	A16V, R18H, C21G, R31C, D45N, P48S, G50S, K67R, V69M, T103A, A118T, P119S
12	*rplP* (L16)	29	10	Q8K, T16I, G26S, A32V, P89T, R93C, A106V, E107K, E123G, A125V
13	*rplQ* (L17)	25	7	H3Y, N11S, R45H, V47A, S62N, A66V, T70I, G81D, D82E, P85S, T88A, A89T, P109S, P119A
14	*rplS* (L19)	29	0	E21K, T38S/I, G56S, E84G, Y101C, A118T
15	*rplT* (L20)	40	2	R11H, H14Y, K16R, R25H, G68C, A69T, S75P, A99V/T, A104V, A119V
16	*rplU* (L21)	23	1	V16I, Q28E, I41V, E45K, M77T, Q87R
17	*rplV* (L22)	26	6	D22N, Q31K, A44V, N57D, H60R, 84-89 ins (KGPSLK), 92-96 ins (QARAK), A99D, E107K
18	*rplX* (L24)	34	1	G30D, K53E, I56L, I57L, M61K, I65L, I65V, N67D, A69S, A78V, I83V, V92I, K93R, F97L
19	*rplY* (L25)	106	2	A51T, T70P, H89Y, D98N, L100P, G123A, L127M, A131T, A131S, L135V, A154V, V172I, V185I, R190C, R190H
20	*rpmA* (L27)	22	1	G13S, S35F, I63V, R77H, V80I
21	*rpmB* (L28)	20	0	R48H, L56S, R72C
22	*rpmE* (L31)	25	10	H6Y, P7L, R10H, V14I, N20H, F22L, G47D
23	*rpmE2* L31-type B	33	43	S18N, A20P, A30T, G31E, G34R, V38A, D41E, G42S, P46L/T, S55A

*Previously reported mutations.

### The Occurrence of HGT in 50S Ribosomal Genes in *N. gonorrhoeae* and Association With Azithromycin MICs

Between 20 and 106 different alleles were found for each of the 23–50S ribosomal proteins median number of alleles 34 [IQR 26–57]. Probable HGT was predicted in three ribosomal genes *rplB*, *rplD*, and *rplY*. *RplB*, and *rplD* genes were assigned to 74 alleles, and the *rplY* gene was assigned to 106 alleles. Unique recombination events were supported by at least 2 out of 7 detection methods. For *rplB* we found that *N. gonorrhoeae* SRR8071393 (allele-71; *n* = 1) was recombinant, and the sequences with the same recombinant event were present in 2,280 *N. gonorrhoeae* isolates and had corresponding minor parent *N. cinerea* NZ_LS483369 (allele-59; *n* = 1; region – 12 to 486 bases) and major parents *N. gonorrhoeae* ERR439316, ERR439342 and ERR775150 (allele-41; *n* = 3; region – 1 to 11 and 487 to 834 nt), which were supported by 6 recombination methods (Table 4 and Supplementary Figure 2A). The gene, *rplD* had *N. gonorrhoeae* ECDC_ES052 (allele-12; *n* = 1) as the recombinant with the same recombination event present in *N. gonorrhoeae* (*n* = 2,320), *N. mucosa* (*n* = 2), *N. lactamica* (*n* = 3), and *N. polysacchareae* (*n* = 1). The minor and major parents were *N. subflava* NZ_CP031251 (allele-53; *n* = 1; region – 350 to 571 nt) and *N. gonorrhoeae* ECDC_GC_095 (allele-14; *n* = 1; region – 1 to 349 and 572–624 nt), respectively. This event was only supported by 2 methods (Table 4 and Supplementary Figure 2B). For *rplY* the recombinant event was supported by 4 methods, and the recombinant was *N. gonorrhoeae* ERR388419 (allele-59; *n* = 1) with the same recombinant event occurring in 10442, 5, and 2 *N. gonorrhoeae*, *N. lactamica*, and *N. mucosa* isolates, respectively. The corresponding minor parent was *N. lactamica* NZ_CP019894 (allele 82, *n* = 1; region – 222 to 370 nt), and the major parent was *N. gonorrhoeae* SRR5827245 (allele-102; *n* = 1; region – 1 – 221 and 371 – 573 nt; Table 4, Supplementary Figure 2C). The start and end breakpoints are provided in Table 4.

**TABLE 4:** Recombinant events for *rplB*, *rplD*, and *rplY.*

Gene/Protein	Recombinant | allele no | Number (n) of isolates | *Neisseria* sps.	Major parent | allele no | Number (n) of isolates | *Neisseria* sps.	Regions derived from Major parent	Minor parent | allele no | Number (n) of isolates | *Neisseria* sps.	Regions derived from Minor parent	Supporting methods
*rplB* (L2)	SRR8071393 | allele-71 | *n* = 1 | Ng	ERR439316 | allele-41 | *n* = 3 | Ng	1–11 and 487–834	NZ_LS4833690 | allele-59 | *n* = 1 | Nc	12–486	GENECONV, Bootscan, MaxChi, Chimera, SiScan, 3Seq
	DRR124721 | allele-6 | *n* = 2273 | Ng			NZ_CP019894 | allele-50 | *n* = 1 | Nl		
	ECDC-FR13-112 | allele-11 | *n* = 1 | Ng			NZ_CP031253 | allele-53 | *n* = 1 | Nl		
	SRR1661246 | allele-60 | *n* = 2 | Ng					
	SRR3349242 | allele-61 | *n* = 1 | Ng					
	SRR3361355 | allele-65 | *n* = 1 | Ng					
	SRR5235170 | allele-66 | *n* = 1 | Ng					
	SRR8559605 | allele-72 | *n* = 1 | Ng					
*rplD* (L4)	ECDC_ES052 | allele-12| *n* = 1| Ng	ECDC_GC_095 | allele-14 | *n* = 1 | Ng	1-349 and 572–624	NZ_CP031251 | allele-53 | *n* = 1 | Ns	350–571	MaxChi, SiScan
	CP028150 | allele-2| *n* = 2| Nmu					
	DRR124721 | allele-5| *n* = 2215| Ng					
	ECDC_MT13_022 | allele-15| *n* = 10| Ng					
	ECDC_RB13001963 | allele-17| *n* = 21| Ng					
	ECDC_SI13_060 | allele-19| *n* = 13| Ng					
	ECDC_T2_ES003 | allele-20| *n* = 3| Ng					
	ERR1426723 | allele-25| *n* = 23| Ng					
	ERR2172259 | allele-27| *n* = 18| Ng					
	ERR3325624 | allele-31| *n* = 1| Ng					
	ERR3325741 | allele-32| *n* = 3| Ng					
	ERR3325826 | allele-33| *n* = 1| Ng					
	ERR3577369 | allele-36| *n* = 2| Ng					
	ERR3578920 | allele-39| *n* = 1| Ng					
	ERR855025 | allele-48| *n* = 1| Ng					
	NC_014752 | allele-50| *n* = 2| Nl					
	NZ_CP019894 | allele-52| *n* = 1| Nl					
	NZ_CP031325 | allele-56| *n* = 1| Np					
	SRR1661281 | allele-61| *n* = 2| Ng					
	SRR3349851 | allele-64| *n* = 1| Ng					
	SRR3350151 | allele-65| *n* = 1| Ng					
	SRR3350241 | allele-66| *n* = 1| Ng					
	SRR7226413 | allele-70| *n* = 1| Ng					
	SRR8071250 | allele-71| *n* = 1| Ng					
*rplQ* (L25)	ERR388419| allele-59| *n* = 1| Ng	SRR5827245 | allele-102 | *n* = 1	1-221 and 371–573	NZ_CP019894 | allele-82 | *n* = 1| Nl	222–370	MaxChi, Chimera, SiScan, 3Seq
	WHO-F| allele-1| *n* = 6223| Ng					
	DRR124706| allele-5| *n* = 449| Ng					
	DRR124721| allele-6| *n* = 3327| Ng					
	DRR124753| allele-7| *n* = 272| Ng					
	DRR124876| allele-8| *n* = 1| Ng					
	ECDC-AT13-524| allele-10| *n* = 47| Ng					
	ECDC-BE13-488| allele-12| *n* = 77| Ng					
	ECDC-FR13-085| allele-15| *n* = 2| Ng					
	ECDC-HU13-088| allele-20| *n* = 10| Ng					
	ECDC-PT13-002| allele-21| *n* = 1| Ng					
	ERR1067822| allele-29| *n* = 1| Ng					
	ERR1426756| allele-34| *n* = 16| Ng					
	ERR2133895| allele-35| *n* = 1| Ng					
	ERR3325552| allele-41| *n* = 1| Ng					
	ERR3325721| allele-43| *n* = 6| Ng					
	ERR3577380| allele-50| *n* = 5| Nl					
	ERR3578067| allele-54| *n* = 1| Ng					
	ERR3578842| allele-56| *n* = 2| Nm					
	ERR363665| allele-58| *n* = 1| Ng					
	ERR388452| allele-60| *n* = 1| Ng					
	ERR439321| allele-64| *n* = 1| Ng					
	ERR439329| allele-65| *n* = 1| Ng					
	ERR779774| allele-74| *n* = 1| Ng					
	ERR976913| allele-78| *n* = 1| Ng					
	Ngono-FA19| allele-80| *n* = 1| Ng					
	SRR3343539| allele-93| *n* = 5| Ng					
	SRR3360647| allele-94| *n* = 1| Ng					
	SRR3360924| allele-97| *n* = 1| Ng					

*For *n* > 1, one representative accession number is provided. Ng, *N. gonorrhoeae*; Ns, *N. subflava*; Nc, *N. cinerea*; Nl, *N. lactamica*; Nm, *N. mucosa*; and Np, *N. polysaccharea.**

Additionally, recombination analysis were carried out for the closely linked *rplD* and *rplB* genes. We found two unique recombination events. The first event was supported by 6 out of 7 detection methods. The major and minor parent were *N. gonorrhoeae* ERR1528156 (region – 1 to 277 and 959 to 1,971 nt) and *N. cinerea* NZ_LS483369 (region – 278 to 958 nt), respectively, with *N. gonorrhoeae* ERR1560943 as the recombinant. The second event was supported by all the 7 methods. *N. gonorrhoeae* ERR155164 was the recombinant, and the sequences with the same recombinant event were present in 180 *N. gonorrhoeae* ECDC isolates and had corresponding minor parent *N. gonorrhoeae* ERR1528156 (region – 1 to 4 and 1,423 to 1,971 nt) and major parent *N. lactamica* NZ_LS483369 (region – 5 to 1,422 nt) and minor parent *N. gonorrhoeae* ERR1528156 (Figure 6). The non-synonymous SNPs in HGT regions of *rplB*, *rplD*, and *rplY* were further investigated to assess the population-wide prevalence and association with azithromycin MIC. Mutations were identified in 16 amino acid positions in L2, 5 in L4 and 1 in L25. The following mutations at amino acids positions R13C, N45H/S, S75P, A112V, V114A, A160V, and D187G/N for L2; A118T, V125A, T130I, A147G, and R157Q for L4; and L100P for L25 were analyzed. All the above mutations except L2 (R13C) and L25 (L100P) were associated with significantly higher azithromycin MICs (Figures 4D–F) across varied genetic backgrounds (Figures 4A–C). Some of these mutations were associated with established macrolide RAMs, meaning the association between the ribosomal protein mutations and elevated MICs may not be causal. Statistically significant associations were found between the presence of C2597T in 23S rRNA and mutations in L2 (N45H/S, S75P, A112V, and V114A) and L4 (A118T, V125A, A147G, and R157Q; all *p* < 0.001; Supplementary Tables 4, 5). Likewise, significant correlations were found between mutations in MtrR (-35A del, A39T, G45D) and both L2 (N45H/S, S75P, and V114A) and L4 (A118T, V125A, A147G, and R157Q, *p* < 0.001; Supplementary Tables 4, 5). Alignments of L2, L4, and L25 alleles are provided as Supplementary Tables 6–8.

**TABLE 5:** Overview of the number of isolates with L2, L4, and L25 HGT events and known AMR mutations.

Mutations		L2 (HGT)					L4 (HGT)					L25 (HGT)				
		No	%	Yes	%	*p*-value	No	%	Yes	%	*p*-value	No	%	Yes	%	*p* value
23S	23S_A2045G	122	97.60%	3	2.40%	<0.0001	122	97.60%	3	2.40%	<0.0001	125	100%	0	0%	<0.001
	23S_C2597T	207	49.52%	211	50.48%	<0.0001	207	49.52%	211	50.48%	<0.0001	410	98.09%	8	1.91%	<0.0001
*mtrR*	−35Adel	4080	98.91%	45	1.09%	<0.0001	4070	98.67%	55	1.33%	<0.0001	4101	99.42%	24	0.58%	<0.0001
	A39T	1982	60.19%	1311	39.81%	<0.0001	2003	60.83%	1290	39.17%	<0.001	2665	80.93%	628	19.07%	<0.0001
	G45D	1191	87.25%	174	12.75%	<0.0001	1190	87.18%	175	12.82%	<0.0001	1192	87.33%	173	12.67%	<0.0001
	G120C	1	100%	0	0%	NS	1	100%	0	0%	NS	0	0%	1	100%	NS
	A121S	1	100%	0	0%	NS	1	100%	0	0%	NS	0	0%	1	100%	NS
	meningitidislikepromoter	7	1.31%	529	98.69%	<0.0001	7	1.31%	529	98.69%	<0.001	536	100%	0	0%	<0.0001
	DistruptedWHO-Plikepromoter	21	100%	0	0%	<0.001	21	100%	0	0%	<0.0001	21	100%	0	0%	NS
L4	G68C	1	100%	0	0%	NS	1	100%	0	0%	NS	1	100%	0	0%	NS
	G68D	39	92.86%	3	7.14%	<0.0001	39	92.86%	3	7.14%	<0.0001	42	100%	0	0%	NS
	G68V	2	100%	0	0%	NS	2	100%	0	0%	NS	2	100%	0	0%	NS
	G70A	8	80%	2	20%	<0.0001	8	80%	2	20%	NS	10	100%	0	0%	NS
	G70D	334	95.70%	15	4.30%	<0.0001	336	96.28%	13	3.72%	<0.0001	337	96.56%	12	3.44%	<0.01
	G70R	2	100%	0	0%	NS	2	100%	0	0%	NS	2	100%	0	0%	NS
	G70S	19	100%	0	0%	<0.001	19	100%	0	0%	<0.001	19	100%	0	0%	NS
	R157Q	34	1.46%	2301	98.54%	<0.0001	0	0	2335	100%	<0.0001	2323	99.49%	12	0.51%	<0.0001

*NS, not significant.*

### Distribution of Horizontally Acquired Ribosomal Genes

A total of 66 (0.56%), 59 (0.5%), and 11,432 (98.17%) isolates belonged to pre-antibiotic (pre-1950s), golden (1950–1970s), and post-modern era (1980s to 21st century), respectively. Eighty-seven (0.74%) isolates did not have any information on the isolation year. Of these, AZM MICs were available for 49 isolates of the pre-antibiotic and golden era and 8988 isolates from the post-modern era (Supplementary Table 9). The proportion of isolates with HGT in *rplB* increased from 7.5% in the pre-antibiotic era to 8.4% in golden era and 20.2% in post-modern era. A similar trend was seen for *rplD* with 1.5%/1.6% in the pre-antibiotic/golden eras to 20.3% in the post-modern era. The opposite trend was observed for isolates with HGT in *rplY* with 51%/50.8% in the pre-antibiotic/golden eras and 9.08% in postmodern era.

**FIGURE 5:**
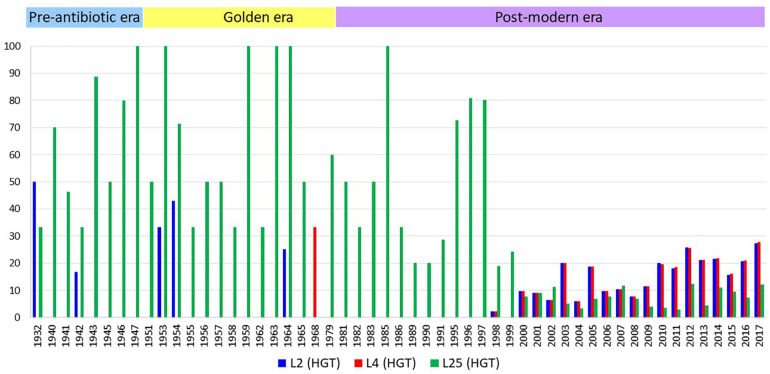
The number of isolates with HGT events per year. *X*-axis represents years, *y*-axis represents the number of isolates in percentage adjusted according to the total number of isolates by year. The number of isolates *n* = 1 and the years with no HGT events are not depicted.

The first observation of gene transfer occurred in 1930 in 1 isolate involving the *rplD* gene, followed by *rplB* gene transfer in 1931 in one isolate and *rplY* gene transfer in 1932 in two isolates (Figure 5 and Supplementary Table 9). In total, 2337, 2355, and 1127 isolates had *rplB*, *rplD*, and *rplY* HGT events, respectively. Out of 68 countries from where the isolates were available, evidence of HGT in *rplB*, *rplD*, and *rplY* were observed in 46 countries.

There was a strong positive correlation between alleles with HGT in L2 and L4 (rho = 0.97; *p* < 0.0001) and a negative correlation between HGT in L25 and L2 (rho = -0.15 *p* < 0.0001), and L25 and L4 (rho = -0.15 *p* < 0.0001). The positive association between HGT in L2 and L4 was most noticeable in ST11428 where 100% of the isolates had HGT in both these loci (*n* = 384) and ST9363 where both L2/L4 HGT were present in 1,163 out of 1,169 isolates (99.5%; Supplementary Table 10). These two sequence types contained 66.4%/65.7% L2/L4 HGT events and had a negligible amount of L25 HGT event (ST9363 – 0.35% and ST11428 – 0%).

All isolates from ST7359 and ST1918 had L25 HGT alleles and together these sequence types comprised 531 out of 1127 L25 HGT alleles (47.1%). These sequence type had no isolates with HGT in L2 or L4. Among the ST9363 isolates (*n* = 1,169), 3 and 87 isolates had the 23S (A2045G) and 23S (C2597T) mutations, respectively. ST11428 (*n* = 384) and ST7359 (*n* = 417) isolates had 23S (C2597T) mutation in 3 and 1 isolates, respectively. Out of 418 isolates, 211 (50.4%) isolates from these two sequence types with the C2597T mutation had L2 or L4 HGT alleles.

Interestingly, almost all of the isolates with HGT in L2 (2301/2335; 98.5%) and L4 (2335/2335; 100%) had the L4 (R157Q) mutation, whereas only 0.5% of isolates with L25 HGT had this L4 mutation (Table 5). The first observation of an isolate with a combined L2 and L4 HGT event was in the year 2001 (ERR1082180, ST1580, AZM MIC – 1 mg/L). This isolate also had 23S (C2597T) and *mtrR* (G45D) mutations but no L25 HGT event.

## Discussion

**FIGURE 6:**
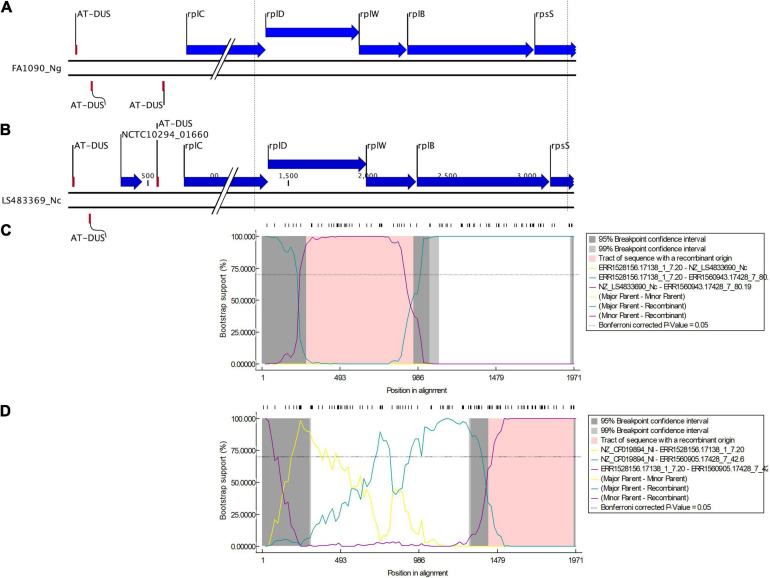
Genetic organization of 50S ribosomal protein in the order from 5’ to 3’: *rplD* (621 bp), *rplW* (321 bp), and *rplB* (834 bp) of **(A)** *N. gonorrhoeae* (FA1090) and **(B)** *N. cinerea* (LS483369). Dotted lines denote the region of sequence used in the multiple alignment. DNA uptake sequence (DUS), here AT-DUS (56’-ATGCCGTCTGAA-3’) is depicted. Bootscan analysis (by RDP4) of the sequences of ECDC (*n* = 1054) and Neisseria commensals (*n* = 15). The *X* axis shows the nucleotide position number and *Y* axis shows the bootstrap support. The dashed line denotes a bootstrap cutoff of 70% **(C)** a recombination event starting at position 278 and ending at position 958 was predicted with *N. gonorrhoeae* ERR1528156 being the major parent isolate and *N. cinerea* NZ_LS483369 being the minor parent isolate **(D)** a recombination event starting at position 1423 and ending at position 1971 was predicted with *N. lactamica* NZ_LS483369 being the major parent isolate and *N. gonorrhoeae* ERR1528156 being the minor parent isolate.

We found evidence of HGT in three gonococcal 50S ribosomal genes *rplB*, *rplD*, and *rplY*. Our analyses suggest that the horizontally transferred DNA was acquired from three commensal *Neisseria* species: *N. cinerea*, *N. subflava*, and *N. lactamica*. In *Neisseria*, the genetic organization of 50S ribosomal protein in the order from 5’ to 3’ is *rplD* (621 bp), *rplW* (321 bp) and *rplB* (834 bp). In order to discern the linkage between *rplD* and *rplB* about 1970 nt including *rplD*, *rplW*, *rplB* and 100 bp downstream and upstream of the genes were analyzed. Recombination with *N. cinerea* generated the *rplD* recombinant, followed by another recombination event with *N. lactamica* generating the *rplB* recombinant. The lengths of the recombined sequence were approximately 678 bases and 544 bases, respectively, which is within the recent estimate (2.5 kb) of the mean of the geometrically distributed DNA tract lengths transferred between donors and recipients in *N. gonorrhoeae* (Arnold et al., 2018; Yahara et al., 2021). It should be noted that the AT-DUS was present in *N. cinerea* NZ_LS483369 at 815 bp upstream of *rplD* (Figure 6).

The transferred DNA contained mutations associated with reduced susceptibility to azithromycin: 7, 5 and 1 mutations in *rplB, rplD*, and *rplY*, respectively. These mutations were, however, also associated with other mutations that are well established determinants of macrolide resistance. Confounding could thus explain the association we observed between these novel mutations in *rplB, rplD, rplY*, and azithromycin MIC. It is also possible that these associations could be explained by the ribosomal mutations compensating for the fitness costs of the RAMs such as those in the 23S rRNA (Melnyk et al., 2015).

The strong association between HGT in L2/L4 and the 23S C2597T mutation could be explained by such a fitness restoring effect. Of note, studies of clinical isolates of *Mycoplasma genitalium* have found associations between the presence of mutations in L4 and L22 and macrolide resistance conferring mutations in 23S rRNA (Cao et al., 2010; Jensen et al., 2014). Likewise, mutations in the S5 and S12 ribosomal protein can confer resistance to spectinomycin and streptomycin, respectively, via structural changes in its rRNA target (Allen and Noller, 1989; Ilina et al., 2013). Mutations in L2 in *Bacillus subtilis* have also been found to confer resistance to bactobolin via altering its interaction with the 23S rRNA (Chandler et al., 2012). Whilst these hypotheses will require experimental validation, recent studies have illustrated the complexity of the effects of ribosomal mutations on bacterial phenotypes. Multiple studies have, for example, found various mutations in L4 and L22 in a range of bacteria, including *N. gonorrhoeae*, leading to macrolide resistance (Gregory and Dahlberg, 1999; Tait-Kamradt et al., 2000; Ma et al., 2020). Many of these studies have assumed that these mutations reduce macrolide susceptibility by narrowing the peptide exit tunnel so that macrolides are not able to bind to the 50S ribosomal subunit (Figures 3A,B) (Moore and Sauer, 2008). However, further experimental evidence in *Escherichia coli* showed that the mechanism was not simple blockage of the exit-tunnel but rather due to reduced intracellular erythromycin concentration via increased expression of AcrAB-TolC efflux pumps. This effect was, in turn, thought to be mediated by increased translation of the relevant mRNAs by reducing programmed ribosome stalling. Similarly, in *Mycobacterium smegmatis*, antibiotic-induced mutations in four ribosomal proteins resulted in large alterations in the transcriptome and proteome, which facilitated the acquisition of mutations in other genes that in turn conferred higher-level resistance (Gomez et al., 2017). These findings suggest that mutations in the ribosomal proteins and their interactions with mutations in other genes (such as the 23S rRNA) may be more important and complex than previously appreciated.

Previous studies in other organisms have found evidence of HGT in the ribosomal proteins, but in all cases, this involved uptake of the entire ribosomal gene (Makarova et al., 2001; Garcia-Vallvé et al., 2002). HGT-mediated chimerization has been previously reported for 16S rRNAs but not for ribosomal proteins (Miyazaki and Tomariguchi, 2019). To the best of our knowledge, this is the first study showing chimerization of ribosomal proteins. This finding provides further support for Miyazaki and Tomariguchi’s “cradle model” of ribosomal evolution which posits that the functionally essential interactions between rRNA and ribosomal proteins (the cradle) were locked-in early on in ribosomal mutation and therefore amenable to HGT events (Miyazaki and Tomariguchi, 2019). Gonococci have a fundamentally non-clonal population structure due to high rates of intraspecies HGT (O’Rourke and Stevens, 1993). In addition, HGT from a number of commensal species has led to mosaicism of a number of gonococcal and meningococcal genes such as *penA* and *mtrCDE* (Bowler et al., 1994; Wadsworth et al., 2018; Yahara et al., 2018). HGT in *rplB*, *rplD*, and *rplY* genes was first detected between 1930 and 1932, which means the original events cannot plausibly be linked with antimicrobial pressure. In fact, the prevalence of L25 HGT alleles declined over time. The increase in the prevalence of isolates with L2/L4 HGT over time could, however, be explained by antimicrobial pressure.

An important limitation of our study is that it only included 15 commensal *Neisseria* isolates and no *N. meningitidis* isolates. The inclusion of a greater number of commensals would be expected to increase the probability of detecting HGT events. Moreover, HGT events that resulted in no or few nucleotide changes would not have been detected by our methodology (Nielsen et al., 2013; Overballe-Petersen et al., 2013). Our methodology also did not allow us to confirm what phenotypic effects the variations in ribosomal proteins had. Nevertheless, we were able to characterize the extensive allelic variations present in Neisserial ribosomal proteins. For the first time, we also established that HGT can contribute to this variation via chimerization of ribosomal proteins between different *Neisseria* species.

## Data Availability Statement

The data we used is publicly available from pathogenwatch and NCBI. The datasets analyzed for this study can be found in the microreact link: https://microreact.org/project/ucwXprtUMAAxXzKyVJwd72, https://microreact.org/project/dxnKsuFhRSAf7BRL6U1cZ3, https://microreact.org/project/hTZbP4fVvNSu3QWLbtaCXf.

## Author Contributions

SSM-B and CK conceptualized the study, interpreted the data, performed the statistical analysis, and wrote the first draft. SSM-B was responsible for data collection, bioinformatic analysis, and Figure 3A. All the authors read and approved the final draft.

## Conflict of Interest

The authors declare that the research was conducted in the absence of any commercial or financial relationships that could be construed as a potential conflict of interest.

## Footnotes

1 https://pathogen.watch/collections

2 https://www.uniprot.org/

## Supplementary Material

Supplementary Figure 1 Global distribution of *N. gonorrhoeae* genomes used in the study. **(A)** Distribution by country. **(B)** Distribution by year.

Supplementary Figure 2 Neighbor-joining trees of **(A)** *rplB* (Major parent: 1–11 nt and 487–834 nt; Minor parent: 12–486 nt; **B)** *rplD* (Major parent: 1–349 nt and 572–624 nt; Minor parent: 350–571 nt) and **(C)** *rplY* (Major parent: 1–221 nt and 371–573 nt; Minor parent: 222–370 nt) nucleotide regions inferred from the major (left) and minor regions (right), showing evidence for the recombination events. All branch lengths are drawn to a scale of nucleotide substitutions per site. The sequences marked with red, salmon red, blue and green colors indicate the recombinant, the sequences with same recombinant event, the minor parent and major parent, respectively. Ng, denotes *N. gonorrhoeae*; Nc, *N. cinerea*; Ne, *N. elongata*; Nf, *N. flavescens*; Nl, *N. lactamica*; Nmu, *N. mucosa*; Ns, *N. subflava*; and Np, *N. polysaccharea*.

Supplementary Figure 3 Statistical significance between **(A)** L1 (T129I), **(B)** L17 (A66V), and **(C)** L4 (G70 D/A/R/S) and their respective wildtype log_2_ AZM MIC distributions as assessed by Mann–Whitney *U* Test. The line inside the box marks the median. The upper and the lower hinges corresponds to the 25th and 75th percentiles. Statistical significance between variants and wildtype MIC distributions are depicted, *p* < 0.0001 in all cases.

Supplementary Table 1 Collections used in the study.

Supplementary Table 2 MLST and azithromycin MIC distribution.

Supplementary Table 3 Molecular characterization of susceptible, intermediate-, and resistant-azithromycin *N. gonorrhoeae* isolates with WT 23S rRNA (*n* = 9527).

Supplementary Table 4 Multivariate correlation probability of horizontally acquired 50 ribosomal gene mutations and the known mutations.

Supplementary Table 5 Kendall’s tau-b and Spearman’s rank correlations.

Supplementary Table 6 Alignment of L2 alleles.

Supplementary Table 4 Alignment of L4 alleles.

Supplementary Table 8 Alignment of L25 alleles.

Supplementary Table 9 Overview of the number of isolates, the period of isolation, and distribution of azithromycin MIC with L2, L4, and L25 HGT events.

Supplementary Table 10 Overview of the number of isolates and ST-types with L2, L4, and L25 HGT events.
