# Supplementary figures and images for: Evidence of Horizontal Gene Transfer of 50S Ribosomal Genes rplB, rplD, and rplY in Neisseria gonorrhoeae
Source: Front Microbiol. 2021 Jun 10;12:683901. doi: 10.3389/fmicb.2021.683901 (PMC8222677; doi:10.3389/fmicb.2021.683901)

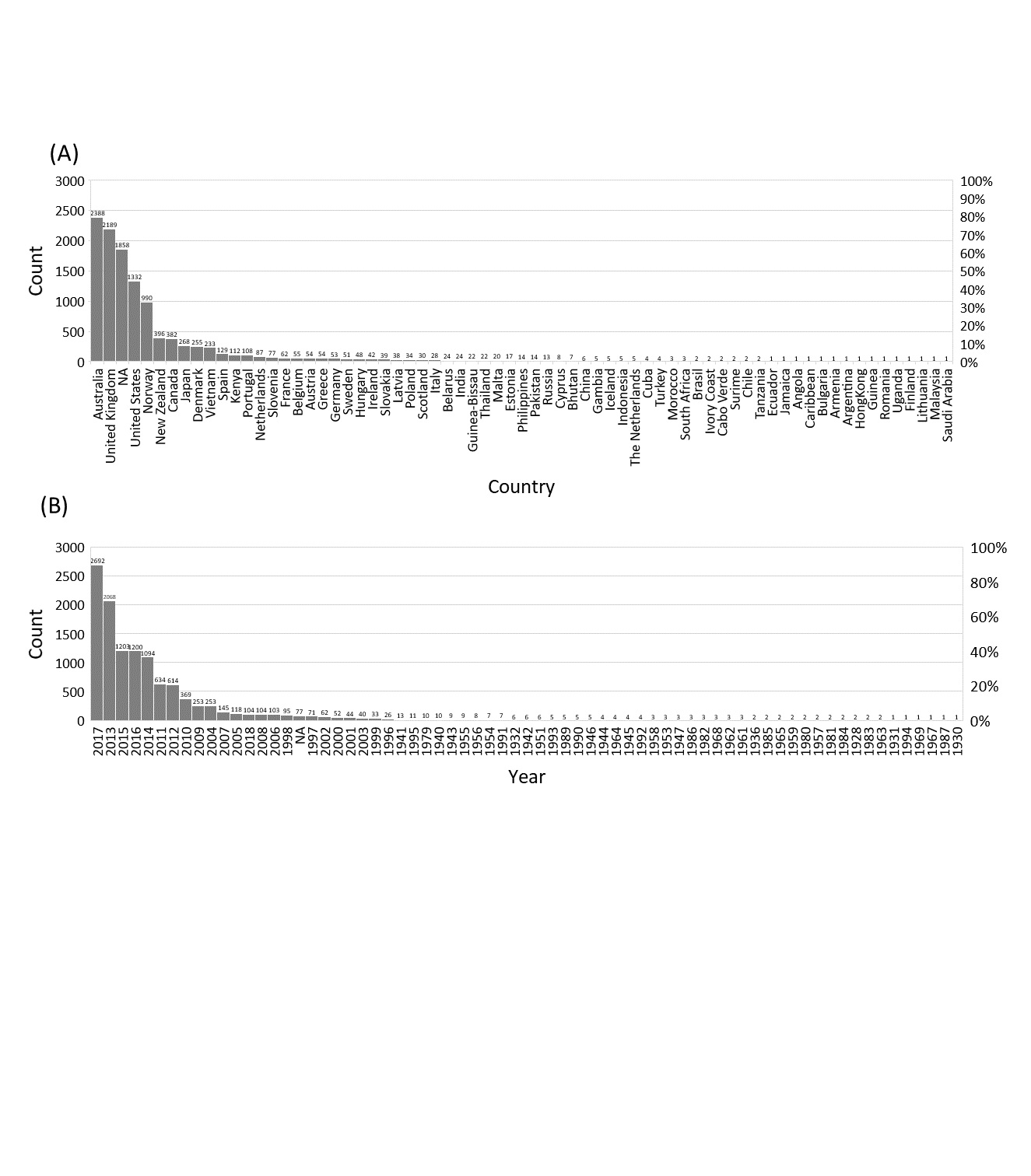

Supplement: Supplementary Figure 1 — Global distribution of N. gonorrhoeae genomes used in the study. (A) Distribution by country. (B) Distribution by year. [file Image_1.JPEG]

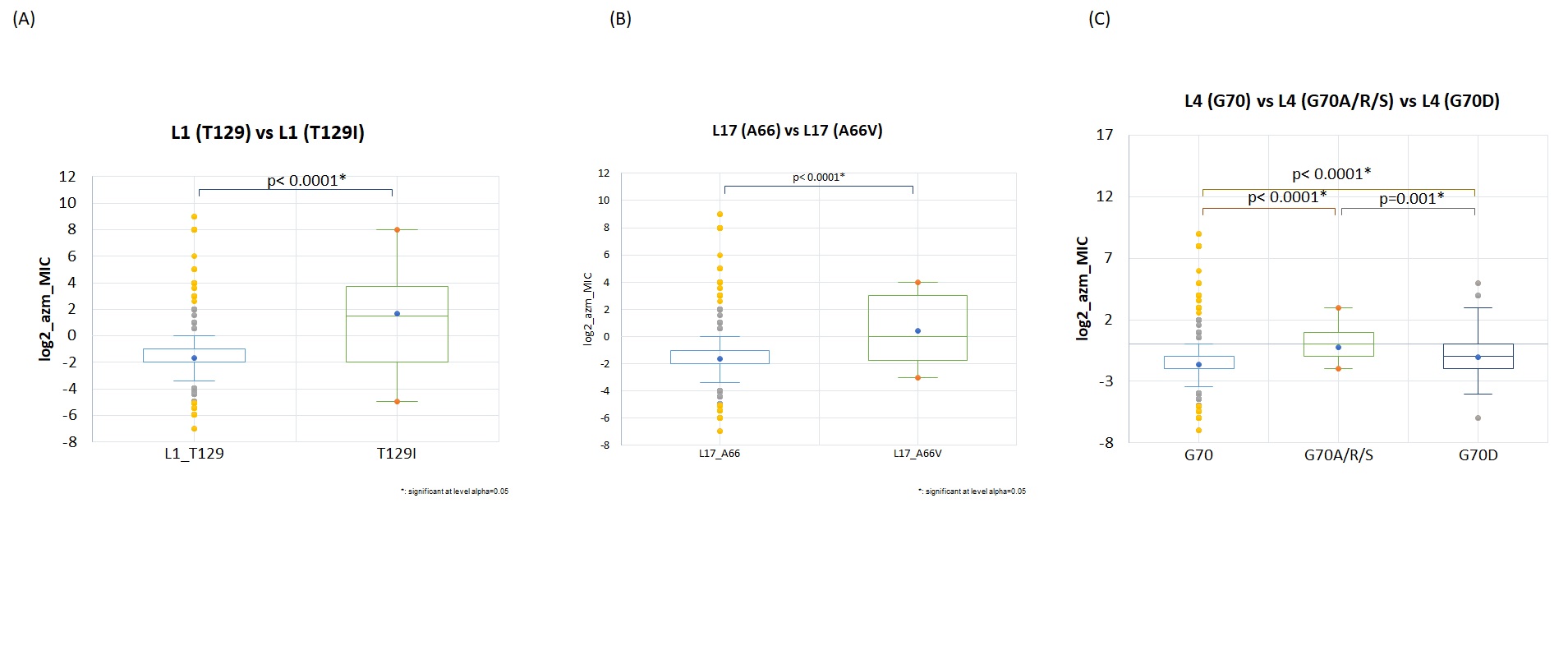

Supplement: Supplementary Figure 3 — Statistical significance between (A) L1 (T129I), (B) L17 (A66V), and (C) L4 (G70 D/A/R/S) and their respective wildtype log2 AZM MIC distributions as assessed by Mann–Whitney U Test. The line inside the box marks the median. The upper and the lower hinges corresponds to the 25th and 75th percentiles. Statistical significance between variants and wildtype MIC distributions are depicted, p < 0.0001 in all cases. [file Image_3.jpeg]
