# Supplementary material for: Evidence of Horizontal Gene Transfer of 50S Ribosomal Genes rplB, rplD, and rplY in Neisseria gonorrhoeae
Source: Front Microbiol. 2021 Jun 10;12:683901. doi: 10.3389/fmicb.2021.683901 (PMC8222677; doi:10.3389/fmicb.2021.683901)
Supplement: Supplementary Figure 2 — Neighbor-joining trees of (A) rplB (Major parent: 1–11 nt and 487–834 nt; Minor parent: 12–486 nt; B) rplD (Major parent: 1–349 nt and 572–624 nt; Minor parent: 350–571 nt) and (C) rplY (Major parent: 1–221 nt and 371–573 nt; Minor parent: 222–370 nt) nucleotide regions inferred from the major (left) and minor regions (right), showing evidence for the recombination events. All branch lengths are drawn to a scale of nucleotide substitutions per site. The sequences marked with red, salmon red, blue and green colors indicate the recombinant, the sequences with same recombinant event, the minor parent and major parent, respectively. Ng, denotes N. gonorrhoeae; Nc, N. cinerea; Ne, N. elongata; Nf, N. flavescens; Nl, N. lactamica; Nmu, N. mucosa; Ns, N. subflava; and Np, N. polysaccharea. [file Image_2.pdf]

(A)

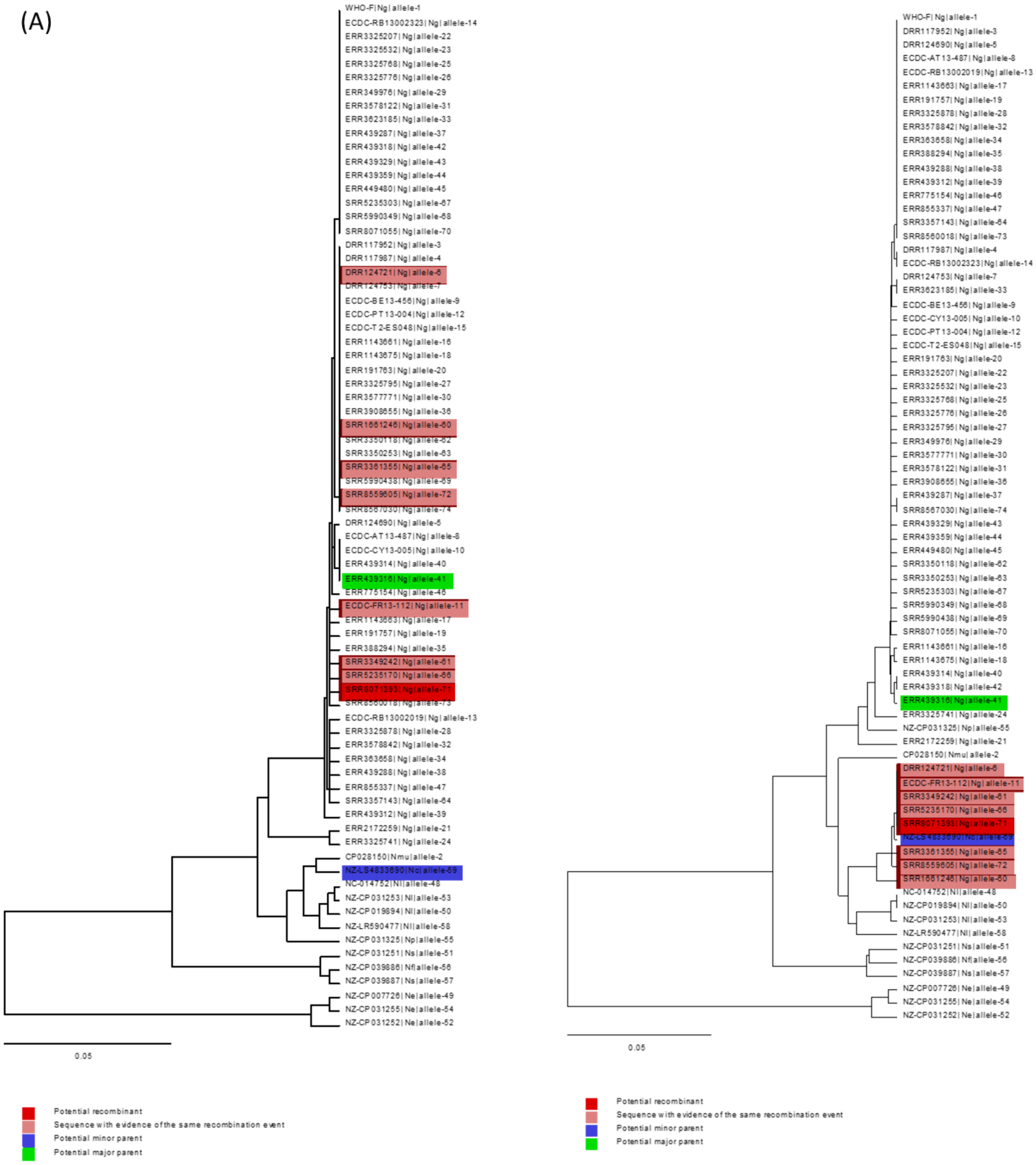

WHO-F|Ng|allele-1
ECDC-RB13002323|Ng|allele-14
ERR3325207|Ng|allele-22
ERR3325532|Ng|allele-23
ERR3325768|Ng|allele-25
ERR3325776|Ng|allele-26
ERR349976|Ng|allele-29
ERR3578122|Ng|allele-31
ERR3623185|Ng|allele-33
ERR439287|Ng|allele-37
ERR439318|Ng|allele-42
ERR439329|Ng|allele-43
ERR439359|Ng|allele-44
ERR449480|Ng|allele-45
SRR5235303|Ng|allele-67
SRR5990349|Ng|allele-68
SRR8071055|Ng|allele-70
DRR117952|Ng|allele-3
DRR117987|Ng|allele-4
DRR124721|Ng|allele-6
DRR124753|Ng|allele-7
ECDC-BE13-456|Ng|allele-9
ECDC-PT13-004|Ng|allele-12
ECDC-T2-ES048|Ng|allele-15
ERR1143661|Ng|allele-16
ERR1143675|Ng|allele-18
ERR191763|Ng|allele-20
ERR3325795|Ng|allele-27
ERR3577771|Ng|allele-30
ERR3908655|Ng|allele-36
SRR1661246|Ng|allele-60
SRR3350118|Ng|allele-62
SRR3350253|Ng|allele-63
SRR3361355|Ng|allele-65
SRR5990438|Ng|allele-69
SRR8559605|Ng|allele-72
SRR8567030|Ng|allele-74
DRR124690|Ng|allele-5
ECDC-AT13-487|Ng|allele-8
ECDC-CY13-005|Ng|allele-10
ERR439314|Ng|allele-40
ERR439316|Ng|allele-41
ERR775154|Ng|allele-46
ECDC-FR13-112|Ng|allele-11
ERR1143663|Ng|allele-17
ERR191757|Ng|allele-19
ERR388294|Ng|allele-35
SRR3349242|Ng|allele-61
SRR5235170|Ng|allele-66
SRR8071393|Ng|allele-71
SRR8560018|Ng|allele-73
ECDC-RB13002019|Ng|allele-13
ERR3325878|Ng|allele-28
ERR3578842|Ng|allele-32
ERR363658|Ng|allele-34
ERR439288|Ng|allele-38
ERR855337|Ng|allele-47
SRR3357143|Ng|allele-64
ERR439312|Ng|allele-39
ERR2172259|Ng|allele-21
ERR3325741|Ng|allele-24
CP028150|Nmu|allele-2
NZ-LS4833690|Nc|allele-59
NC-014752|Nl|allele-48
NZ-CP031253|Nl|allele-53
NZ-CP019894|Nl|allele-50
NZ-LR590477|Nl|allele-58
NZ-CP031325|Np|allele-55
NZ-CP031251|Ns|allele-51
NZ-CP039886|Nf|allele-56
NZ-CP039887|Ns|allele-57
NZ-CP007726|Ne|allele-49
NZ-CP031255|Ne|allele-54
NZ-CP031252|Ne|allele-52
0.05
Potential recombinant
Sequence with evidence of the same recombination event
Potential minor parent
Potential major parent
WHO-F|Ng|allele-1
DRR117952|Ng|allele-3
DRR124690|Ng|allele-5
ECDC-AT13-487|Ng|allele-8
ECDC-RB13002019|Ng|allele-13
ERR1143663|Ng|allele-17
ERR191757|Ng|allele-19
ERR3325878|Ng|allele-28
ERR3578842|Ng|allele-32
ERR363658|Ng|allele-34
ERR388294|Ng|allele-35
ERR439288|Ng|allele-38
ERR439312|Ng|allele-39
ERR775154|Ng|allele-46
ERR855337|Ng|allele-47
SRR3357143|Ng|allele-64
SRR8560018|Ng|allele-73
DRR117987|Ng|allele-4
ECDC-RB13002323|Ng|allele-14
DRR124753|Ng|allele-7
ERR3623185|Ng|allele-33
ECDC-BE13-456|Ng|allele-9
ECDC-CY13-005|Ng|allele-10
ECDC-PT13-004|Ng|allele-12
ECDC-T2-ES048|Ng|allele-15
ERR191763|Ng|allele-20
ERR3325207|Ng|allele-22
ERR3325532|Ng|allele-23
ERR3325768|Ng|allele-25
ERR3325776|Ng|allele-26
ERR3325795|Ng|allele-27
ERR349976|Ng|allele-29
ERR3577771|Ng|allele-30
ERR3578122|Ng|allele-31
ERR3908655|Ng|allele-36
ERR439287|Ng|allele-37
SRR8567030|Ng|allele-74
ERR439329|Ng|allele-43
ERR439359|Ng|allele-44
ERR449480|Ng|allele-45
SRR3350118|Ng|allele-62
SRR3350253|Ng|allele-63
SRR5235303|Ng|allele-67
SRR5990349|Ng|allele-68
SRR5990438|Ng|allele-69
SRR8071055|Ng|allele-70
ERR1143661|Ng|allele-16
ERR1143675|Ng|allele-18
ERR439314|Ng|allele-40
ERR439318|Ng|allele-42
ERR439316|Ng|allele-41
ERR3325741|Ng|allele-24
NZ-CP031325|Np|allele-55
ERR2172259|Ng|allele-21
CP028150|Nmu|allele-2
DRR124721|Ng|allele-6
ECDC-FR13-112|Ng|allele-11
SRR3349242|Ng|allele-61
SRR5235170|Ng|allele-66
SRR8071393|Ng|allele-71
NZ-LS4833690|Nc|allele-59
SRR3361355|Ng|allele-65
SRR8559605|Ng|allele-72
SRR1661246|Ng|allele-60
NC-014752|Nl|allele-48
NZ-CP019894|Nl|allele-50
NZ-CP031253|Nl|allele-53
NZ-LR590477|Nl|allele-58
NZ-CP031251|Ns|allele-51
NZ-CP039886|Nf|allele-56
NZ-CP039887|Ns|allele-57
NZ-CP007726|Ne|allele-49
NZ-CP031255|Ne|allele-54
NZ-CP031252|Ne|allele-52
0.05
Potential recombinant
Sequence with evidence of the same recombination event
Potential minor parent
Potential major parent

(B)

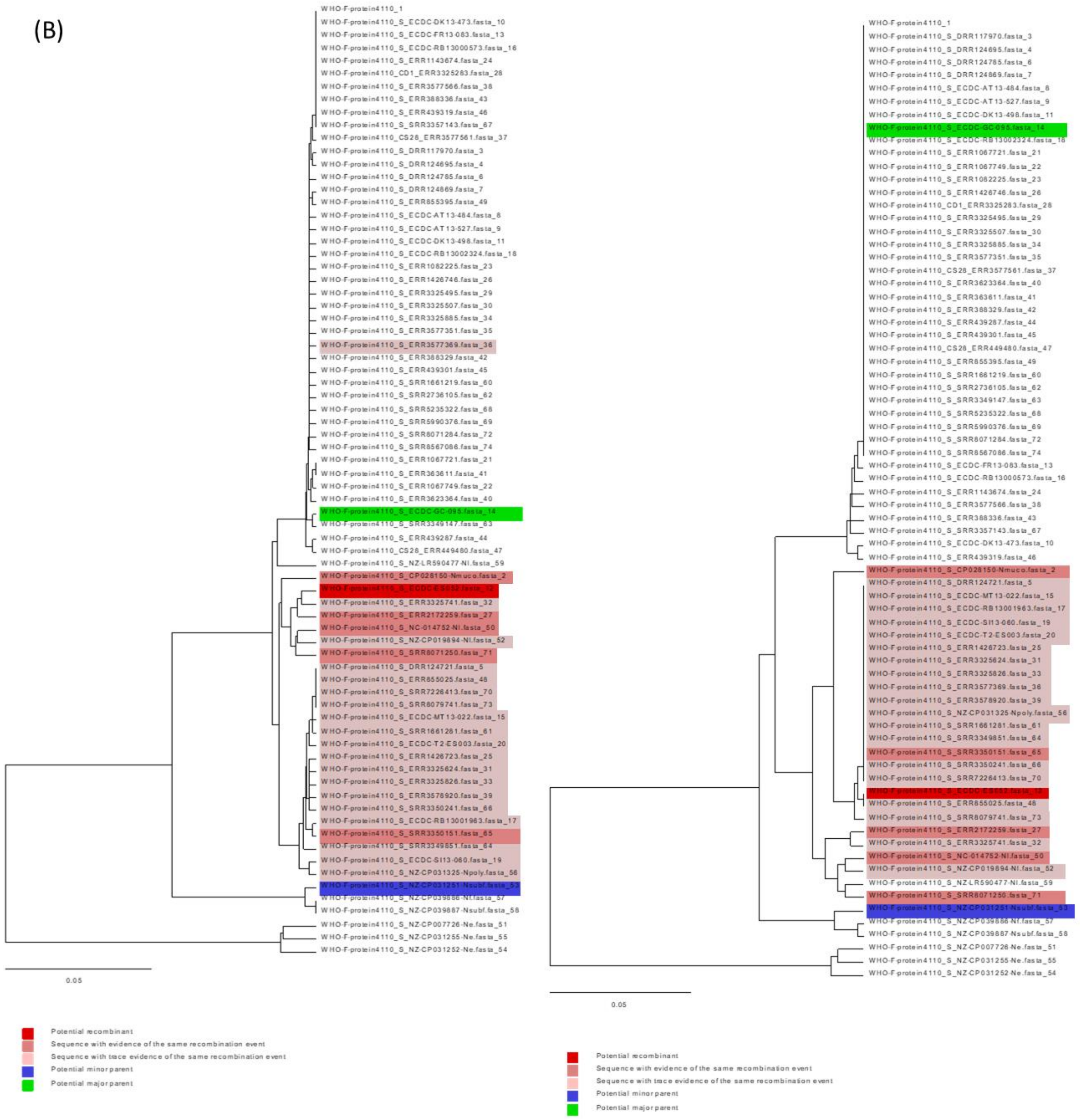
0.05
0.05
Potential recombinant
Sequence with evidence of the same recombination event
Sequence with trace evidence of the same recombination event
Potential minor parent
Potential major parent

(C)

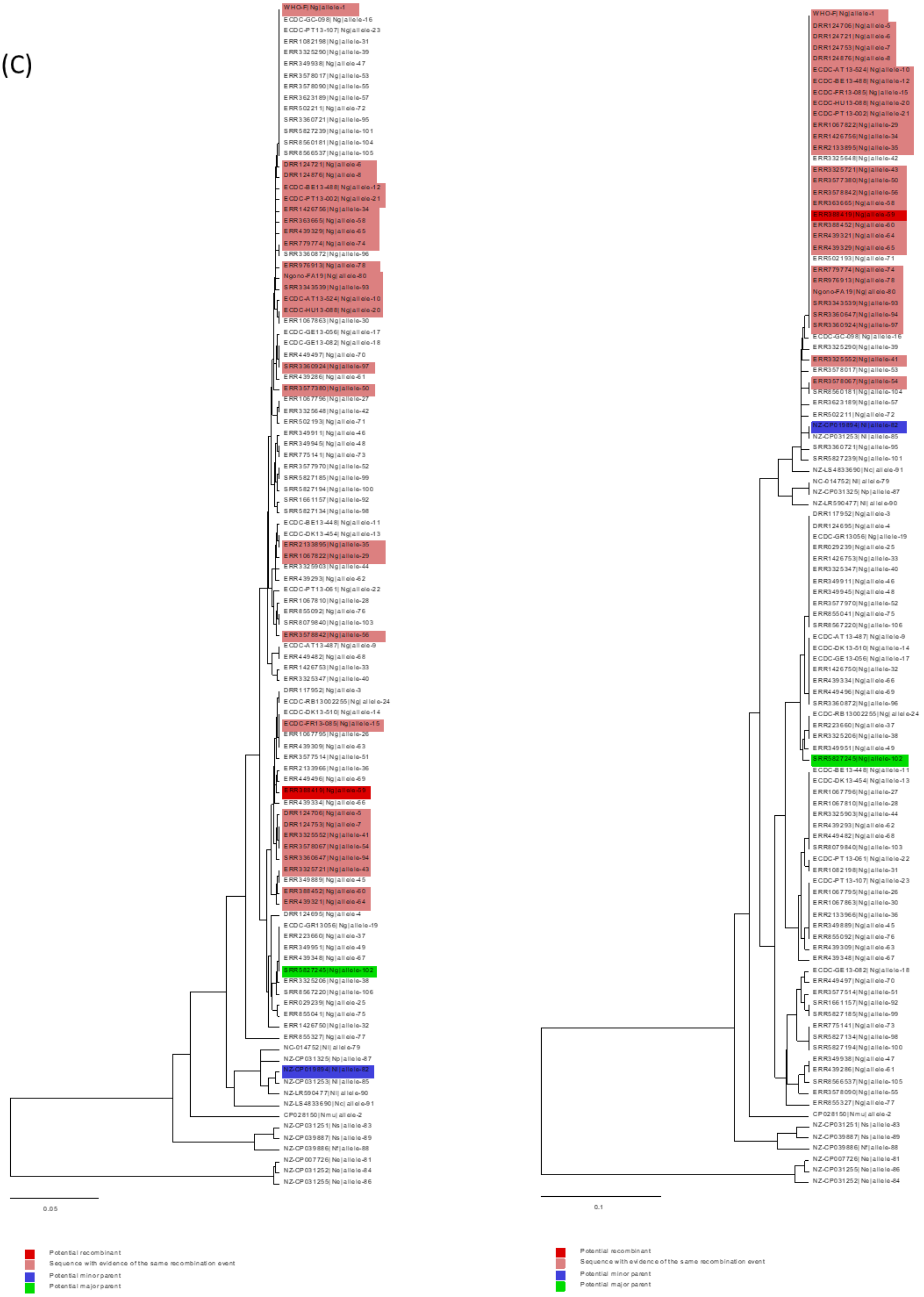
0.05
Potential recombinant
Sequence with evidence of the same recombination event
Potential minor parent
Potential major parent
0.1
Potential recombinant
Sequence with evidence of the same recombination event
Potential minor parent
Potential major parent
